# Random Migration and Signal Integration Promote Rapid and Robust T Cell Recruitment

**DOI:** 10.1371/journal.pcbi.1003752

**Published:** 2014-08-07

**Authors:** Johannes Textor, Sarah E. Henrickson, Judith N. Mandl, Ulrich H. von Andrian, Jürgen Westermann, Rob J. de Boer, Joost B. Beltman

**Affiliations:** 1 Theoretical Biology & Bioinformatics, Utrecht University, Utrecht, The Netherlands; 2 Department of Pathology, Harvard Medical School, Boston, Massachusetts, United States of America; 3 Lymphocyte Biology Section, National Insitutes of Health, Bethesda, Maryland, United States of America; 4 Institute for Anatomy, University of Lübeck, Lübeck, Germany; 5 Division of Immunology, The Netherlands Cancer Institute, Amsterdam, The Netherlands; National Institute of Health (NIH), United States of America

## Abstract

To fight infections, rare T cells must quickly home to appropriate lymph nodes (LNs), and reliably localize the antigen (Ag) within them. The first challenge calls for rapid trafficking between LNs, whereas the second may require extensive search within each LN. Here we combine simulations and experimental data to investigate which features of random T cell migration within and between LNs allow meeting these two conflicting demands. Our model indicates that integrating signals from multiple random encounters with Ag-presenting cells permits reliable detection of even low-dose Ag, and predicts a kinetic feature of cognate T cell arrest in LNs that we confirm using intravital two-photon data. Furthermore, we obtain the most reliable retention if T cells transit through LNs stochastically, which may explain the long and widely distributed LN dwell times observed *in vivo*. Finally, we demonstrate that random migration, both between and within LNs, allows recruiting the majority of cognate precursors within a few days for various realistic infection scenarios. Thus, the combination of two-scale stochastic migration and signal integration is an efficient and robust strategy for T cell immune surveillance.

## Author Summary

Each of the immune system's T lymphocytes recognizes a highly specific molecular pattern, and only a very few T cells are capable of detecting any given infection. These rare cells are at first scattered throughout the body when a pathogen invades the host. To mount an immune response, they need to come together within lymphatic tissue near the infection site, and find cells that carry molecular fragments of the pathogen. Remarkably, experiments show that the immune system solves this needle-in-a-haystack problem in just a few days for various bacterial and viral infections. Aiming to understand how the immune system achieves this, we built a model that brings together classic and recent data on T cell migration. Our model explains how perpetual migration of T cells *between* and *within* lymphatic organs helps to find invading pathogens swiftly and reliably. Specifically, our results suggest that T cells can collect signals from activation-inducing cells for several hours, which allows for reliable detection of even low-profile infections. Thus, random T cell trafficking between and within lymphatic organs robustly protects against a broad range of pathogens, and comes close to an “optimal” surveillance strategy.

## Introduction

Pathogens are enormously diverse. They differ in tissue localization, epitope expression, virulence, and many other factors. Still, our immune system has to swiftly cope with invading pathogens to ensure our survival. Intriguing evidence from rather different infection models like influenza (a local infection of the respiratory tract), dermal herpes simplex, and listeriosis (a systemic infection) shows that the immune system manages to activate a majority of the Ag-specific T cell precursors within just a few days [1], [2]. How can this remarkable efficiency and robustness be achieved?

A key component of our immune system's defense strategy is to keep T cells and other lymphocytes constantly mobile. Because the T cell repertoire needs to be both specific and diverse, each T cell recognizes only a few epitopes. Conversely, only very few T cells – in mice, as little as 20–200 [3]–[5] – can respond to any given Ag. To avoid that local pathogen intrusions go unnoticed, T cells search for Ag proactively by migrating *between* and *within* different organs and tissues. Lymphocyte migration between tissues has been studied for decades, notably from the 1960s to the 1980s [6], whereas cell migration within tissue has become amenable to experiments only recently with the advent of two-photon imaging [7], [8]. Here, we combine classic and recent data about T cell migration on both scales into a common model. Our goal is to pinpoint the key aspects of T cell trafficking that help the immune system respond firmly and rapidly against many different pathogens.

Several previous modeling studies have addressed individual aspects of T cell migration in their own right, many of them spurred by pioneering intravital two-photon experiments that surprisingly showed lymphocyte migration in LNs to be random-walk-like [9], [10]. These models have provided insights into stop-and-go T cell motion [11], the relationship between LN transit time and LN structure [12], [13], and the time needed for T cells to find dendritic cells (DCs) presenting cognate Ag [11], [14], [15]. Fewer models have addressed LN migration between organs [16]–[19], and only recently have the first models combined between-organ migration with a simple representation of T cell priming in LNs as an exponential decay process [20], [21]. From two-photon imaging, we know however that T cell priming in LNs follows a more complex three-phase timecourse [22], [23]. Here we combine existing hypotheses on T cell priming to build a general kinetic model of T cell retention in LNs. Fitting our model against imaging data suggests that T cells in LNs can integrate Ag signals on a timescale of hours, which might help to detect even low-dose Ag reliably. Moreover, we combine the priming kinetics with an explicit model of T cell migration within and between LNs, blood and spleen to ask how two-scale migration and priming interact and affect each other. Specifically, we study the impact of signal integration on the trade-off between fast recirculation and thorough Ag search [20], [21], and ask why *in vivo* LN transit times are so broadly distributed. Finally, we show that the fast T cell recruitment observed *in vivo* for various infections [1], [2] can indeed be explained by two-scale stochastic migration.

## Results

### Signal integration implies a switch-like T cell retention timecourse

**Figure 1:**
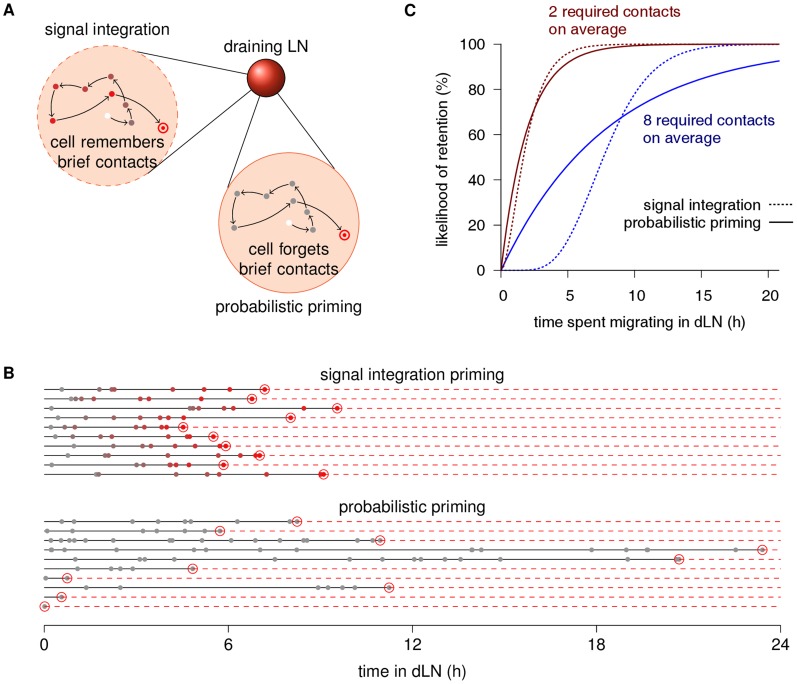
Signal integration leads to switch-like retention kinetics. **(A)** Illustration of signal integration and probabilistic priming in a dLN. With signal integration, the cell remembers each brief DC contact (small circles) on its path (arrows), and retention (double circle) occurs after an Ag-dependent number of contacts (here, 8). With probabilistic priming, retention occurs upon each contact with an Ag-dependent probability (here, 1/8), and otherwise, the contact is instantly forgotten. **(B)** Example simulation trajectories of signal integration and probabilistic priming. In both cases, the waiting times between DC encounters are exponentially distributed with an average waiting time of 1 h. The additional stochasticity in the probabilistic priming leads to a wider distribution of retention times. **(C)** Retention kinetics for signal integration (dashed lines) and probabilistic priming (solid lines). Like in (B), *in silico* cells encounter one cognate DC per hour on average. Ag-dependent parameters (left red lines: 2 required contacts for signal integration and 1/2 success probability for probabilistic priming; right blue lines: 8 contacts, 1/8 success probability) are set such that the average retention times are 2 h (red) and 8 h (blue).

T cell priming in LNs can occur in 3 distinct phases [22], [24], [25]: In phase I, the T cell remains motile and establishes serial brief contacts (lasting a few minutes) with Ag-bearing DCs until, in phase II, the cell comes to a halt and establishes a stable DC contact (lasting hours). Ultimately, in phase III, it detaches from the DC, migrates away, and starts proliferating. T cells upregulate CD69 during phase I [22], suggesting that the brief contacts are immunologically productive and allow to integrate Ag signals from several DCs before committing to retention. Alternatively, given that Ag dose may vary among DCs, T cells in phase I could simply fail to find a DC with a high enough dose for retention, thus the brief contacts might represent unsuccessful retention attempts that do not contribute to reaching phase II. The latter hypothesis has been termed *probabilistic priming* [26]. Despite recent advances that allow to combine intravital cell tracking with *in situ* cytometry [27]–[29], demonstrating that signal integration occurs *in vivo* remains difficult because phase I lasts for several hours, and it is currently infeasible to track single cells that long in intravital imaging experiments [30]. We therefore used a mathematical model to derive testable predictions from the signal integration and probabilistic priming hypotheses (Figure 1A; Methods), and tested these predictions against *in vivo* two-photon data.

Because T cell migration through LN tissue resembles a persistent random walk [13], [31], we considered waiting times between DC encounters to be exponentially distributed, a simplification that has been used and validated in a similar sphere model of T cell random walk in LNs [15]. With probabilistic priming, the waiting time is interpreted as the time required to find a *new* DC, as multiple encounters with the same DC that does not present enough peptide do not contribute to retention. For each new contact, the DC presents a sufficient amount of peptide with a probability that depends on the Ag. For example, at a 1/8 probability, retention occurs on average after 8 unique contacts. With signal integration, multiple contacts with the same DC (or different DCs) do contribute to retention, which occurs after an Ag-dependent number of contacts. For persistent random walks in a relatively large three-dimensional structure like a LN, one can expect roughly 2/3rd of all contacts to be unique due to Polya's recurrence theorem [32]. This expectation has been confirmed by a detailed agent-based model of T cell–DC contacts in lymph nodes [11]. Hence, at the same “true” underlying DC contact rate, the effective contact rate of probabilistic priming is about 2/3rd that of signal integration. In the rest of this paper, we only refer to the effective contact rate for each model.

With both priming models, the time until retention (duration of phase I) is stochastic due to the waiting times, and the variance of this duration differs between the models. For instance, when comparing simulation trajectories of both models at (on average) 8 required contacts and the same effective contact rate, retention typically starts earlier, but completes later with probabilistic priming than with signal integration (Figure 1B). In other words, probabilistic priming implies gradual retention, whereas signal integration leads to a switch-like timecourse (Figure 1C, blue lines). This observation is independent of the contact rate, which equally affects the time scaling of both models. However, at higher Ag doses, the difference between the 2 priming models is much smaller (Figure 1C, red lines), because signal integration becomes less relevant when retention can occur after 1 or a few contacts. Nevertheless, this basic effect implies that signal integration completes retention of an entire Ag-specific cell population faster as well as more reliably than probabilistic priming.

### Extracting T cell retention kinetics from two-photon data

**Figure 2:**
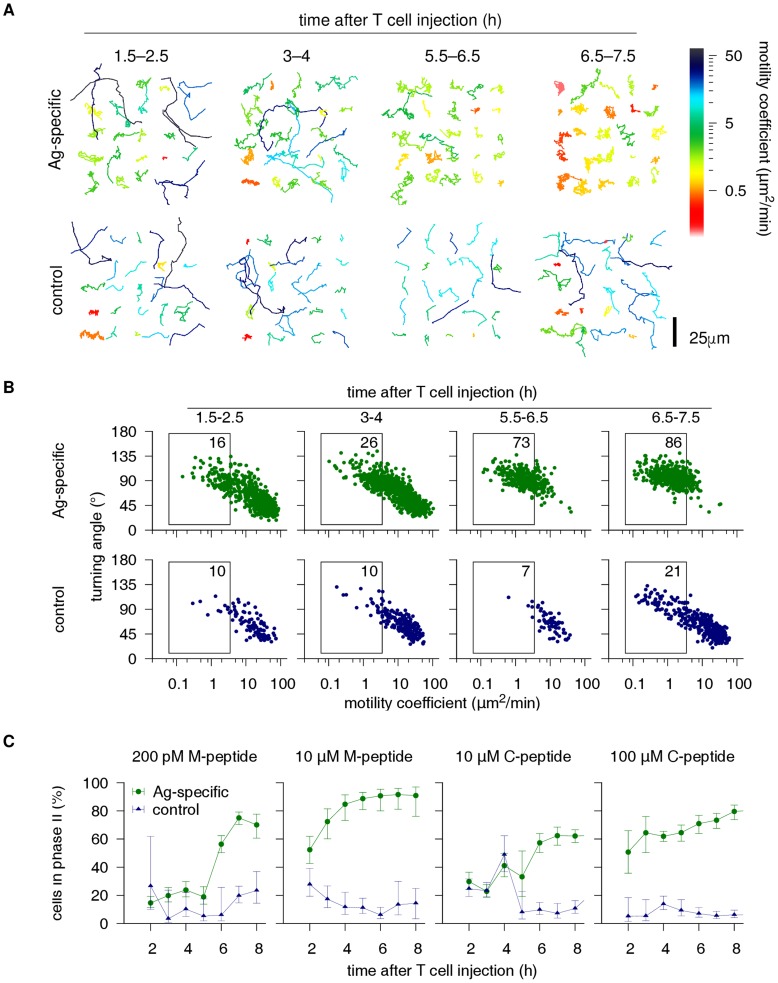
Extracting T cell retention kinetics from two-photon data. **(A)** Tracks of cognate (top) and control (bottom) T cells imaged at different times after simultaneous entry within LNs containing DCs pulsed with 200 pM of M-peptide (see text; ref. [25]). For each video, 25 tracks are shown aligned on a 5×5 grid. Each track is colored according to its motility coefficient, revealing a marked motility reduction of the Ag-specific cells. **(B)** “FACS-like” approach to quantifying cell retention kinetics. Each dot represents a cell track, and each plot shows all tracks from 1 video. Gating is used to define retained cells. Numbers indicate the percentage of tracks within each gate. The plots confirm the qualitative observation of (A) that the motion of Ag-specific cells becomes slower over time (lower motility coefficients). Moreover, persistence decreases (higher turning angles). **(C)** Aggregate results of the gating approach shown in (B) applied to 32 two-photon videos from 10 independent experiments [25]. For each Ag configuration (described in main text), tracks from all videos were pooled and then grouped in 1 h bins. Dots and error bars show means and bootstrapped 95% confidence intervals for the fractions of retained cells. For both peptides, low-dose retention kinetics exhibit a switch pattern similar to that predicted by signal integration priming (Figure 1C, dashed lines).

The switch-like retention kinetics predicted by signal integration (Figure 1C) provide a testable prediction that can be confirmed or rejected by experiments. To determine the retention kinetics of real T cells, we applied a “FACS-like” motility analysis [27] to a set of ∼22,000 T cell tracks extracted from 38 two-photon videos from a previous study [25] where naive Ag-specific and control T cells were imaged at different time points after synchronized entry into popliteal LNs containing peptide-pulsed DCs (Figure 2A). In these experiments, varying doses of 2 peptides were used that differed only in the terminal MHC anchor residue (“M-peptide” with high MHC affinity, or “C-peptide” with low affinity [25]). We estimated the fraction of retained cells in each video by “gating” T cells on the motility coefficient estimated from their track (Figure 2B; Methods), which confirmed that retention increased over time for the Ag-specific but not for the control cells (Figure 2C). At high Ag doses, most T cells were retained early on (Figure 2C, 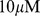 M-peptide and 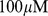 C-peptide). They should therefore have entered phase II after only a few contacts, making it difficult to assess whether signal integration took place (cf. Figure 1C). However, at low Ag doses (Figure 2C, 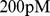 M-peptide and 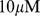 C-peptide), retention kinetics were indeed markedly switch-like, as predicted by our model. Specifically, most cell retention occurred at 4–5 h after cell transfer, whereas most retention should occur shortly after LN entry with probabilistic priming. These data suggest that cells integrate signals *in vivo* during phase I on a timescale of hours, which governs the onset of phase II.

### Statistical analysis of *in vivo* retention kinetics

**Figure 3:**
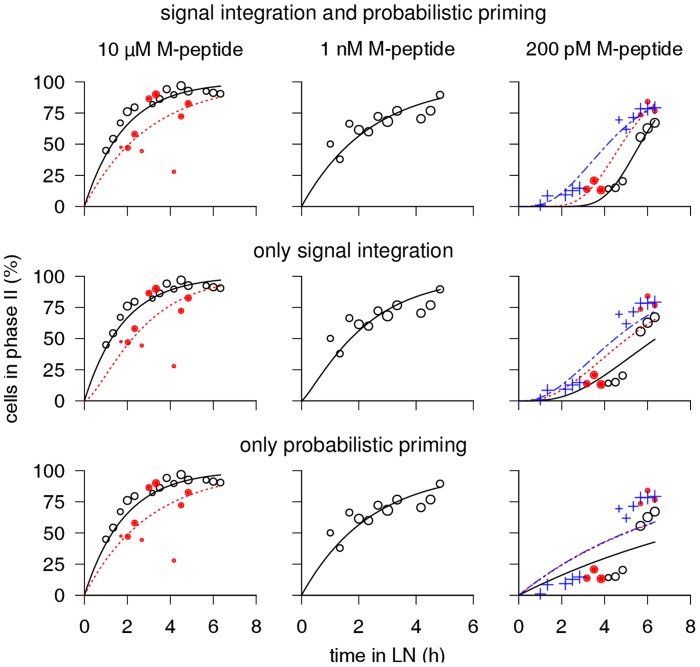
Quantifying the contribution of signal integration and probabilistic priming to *in vivo* T cell retention kinetics. Starting with a general model that accounts for both signal integration and probabilistic priming (top row), we disable each model component separately by setting its parameter to a constant value. Specifically, fixing the success rate to 100% disables probabilistic priming by enforcing that, during every contact, a (possibly small) cognate signal is transmitted (middle row). Similarly, fixing the required contacts to 1 disables signal integration (bottom row). The best fit of each model to retention timecourses obtained from the two-photon data for the M-peptide are shown. In contrast to the coarser analysis of Figure 2, we now consider each experiment separately (filled circles, open circles, and crosses) and use a higher time resolution (details in Methods). The size of each data symbol is proportional to the total duration of all tracks it represents. The characteristic switch-like pattern of signal integration is clearly present in the 200 pM dose (right panels), which the purely probabilistic model fails to fit.

To more precisely quantify the level of support that our data lends to the signal integration hypothesis, we employed statistical model selection starting from a general model that accommodates both signal integration and probabilistic priming (Methods). The 3 parameters of this general model are as follows. First, T cells encounter DCs at a fixed rate. Second, there is a peptide dose-dependent success probability for each contact, with “success” meaning that a cognate signal is transmitted. Third, there is a dose-dependent number of successful contacts required for T cell retention. Because the C-peptide has a very short half-life of 2.4 h on the MHC molecule compared to 6 h for the M-peptide [25], implying that T cell priming might stop within the 8 h time frame of interest, we first analyzed the M-peptide data only. Specifically, we fitted the general model to six M-peptide datasets comprising 3 different Ag doses (Figure 3). Each dataset was recorded in an independent experiment and consisted of 2 or 3 two-photon videos imaged at different times upon cell entry. We constrained the underlying DC encounter rate to be equal across all videos, as the number of injected DCs was constant. Further details on the fitting procedure can be found in the Methods. The general model (top row of Figure 3) gave an acceptable fit and showed good agreement among different experiments with the same Ag dose.

Next, we created 2 restricted versions of our general model by disabling signal integration or probabilistic priming, which leads to the “pure” signal integration and probabilistic priming models shown in Figure 1C. By comparing the Bayesian information criterion (BIC) score of each pure model fit (Figure 3, middle and bottom rows) to the general model fit (Figure 3, top row), we assessed the relative importance of each priming mechanism in the general model. The general model fits the data best in terms of BIC, suggesting that both priming mechanisms are required for explaining the data. However, the BIC score (misfit) increased by 24.6 when signal integration was disabled (purely probabilistic priming) but only by 1.41 when probabilistic priming was disabled (pure signal integration). Applying a common interpretation scale for BIC [33], this indicates that the evidence for probabilistic priming is quite weak (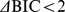) whereas the evidence for signal integration is very strong (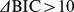). Similar results were obtained when fitting the models to the M-peptide and C-peptide data combined (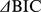 to general model: 0.6 without probabilistic priming and 20.2 without signal integration). However, the model fit to the C-peptide data was considerably poorer (not shown), probably due to the rapid peptide loss which the model does not take into account.

Overall, our statistical analysis lends further support to the hypothesis that T cells integrate signals from DCs they encounter. For high Ag doses this is difficult to distinguish from probabilistic priming because only few interactions lead to T cell retention, yet at low Ag doses the signal integration is clearly detectable.

### Constructing a two-scale model of T cell migration

**Figure 4:**
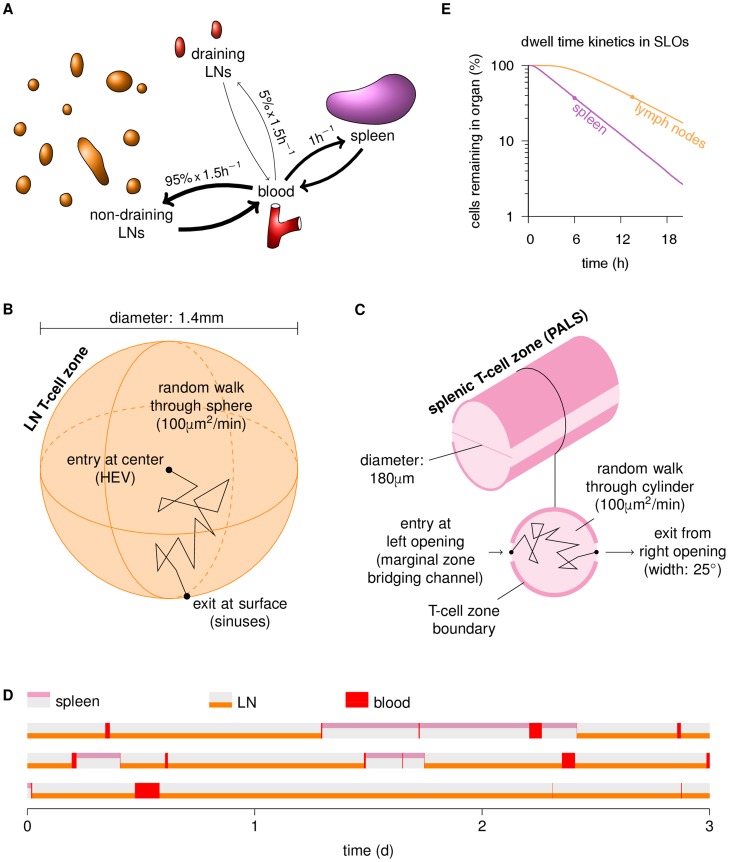
Schematic overview of our stochastic two-scale migration model. **(A)** We consider circulation of Ag-specific T cells in mice between blood and T cell zones in lymph nodes (LNs) and spleen. The number of LNs is set to 30 [36], and when simulating infections we distinguish between draining LNs (dLNs) and non-draining LNs (ndLNs). From the blood, T cells enter into LNs and spleen at different rates (Table 1) – e.g., with a blood-to-LN homing rate of 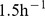, the number of cells entering LNs per hour is 1.5-fold larger than the number of cells in the blood. While dLN homing rates are typically small (e.g. 5% of the total LN homing rate), they can increase over time. **(B)** The transit through LNs is modeled as a random walk through a 3D sphere, where the cell starts in the center and exits back into the blood upon reaching the surface. **(C)** T cell zones in the spleen are represented as cylinders where cells enter at an aperture on the left side. In contrast to the LN, cells cannot penetrate the cylinder surface except through an aperture on the right side, from where they exit. **(D)** Trajectories of 3 simulated cells, illustrating the stochasticity of the migration pattern. For instance, in the first trajectory, the cell starts in a LN until, at ∼9 h, it recirculates to the blood where it resides for ∼30 min. Then it homes to a LN where it dwells ∼22 h, briefly visits the blood at ∼31 h, enters the spleen where it stays for ∼10 h, and continues circulating. **(E)** Cell egress kinetics from LN and spleen resulting from the geometrical parameters and the motility coefficient shown in (B,C): The resulting mean transit times (circles) are 6 h for the spleen and 13.5 h for the LNs, matching both classic [37], [65], [66] and recent estimates [35]. The transit time distribution resembles the exponential distribution used in a recent T cell migration model [21], which would yield a straight line on this plot. However, our *in silico* cells have to traverse the distance from the entry to the exit locations, and therefore only start exiting after a “lag time” of a few hours, rather than immediately after they enter.

To study the interplay between priming within LNs and trafficking between LNs, we designed a stochastic two-scale model of T cell trafficking between secondary lymphoid organs (SLOs) in mice (Figure 4), similar to previous models [17], [20], [21] but anatomically more explicit. In the new model, cells in the blood home to T cell zones in LNs and splenic white pulp (Figure 4A). We represent the T cell areas in the LN paracortex as three-dimensional spheres (Figure 4B), which *in silico* cells enter in the center and then migrate randomly until reaching the surface. The sphere center represents a high endothelial venule, and the surface represents cortical sinusoids as well as subcapsular and medullary sinuses. In contrast, splenic T cell areas (periarteriolar lymphoid sheaths, or PALS) are cord-like structures around central arterioles in the white pulp, which T cells are thought to access via so-called marginal zone bridging channels [34]. In our model we represent the PALS as a cylinder with small apertures on both sides, with 1 aperture being used for entry and the other for exit (Figure 4C). The length of this cylinder is irrelevant for our purpose, because movement along the cylindrical main axis does not bring the cell closer to or further away from an exit site.

**Figure 5:**
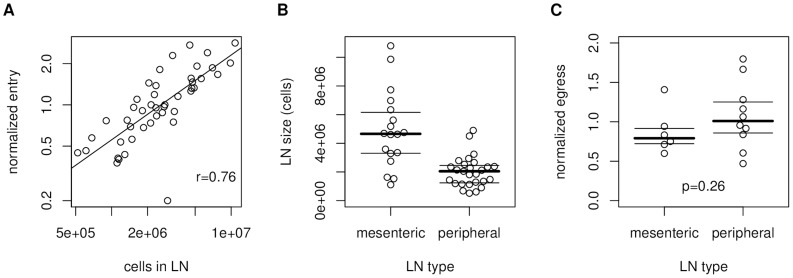
LN size governs T cell entry rate, but not egress rate. (A) Correlation between the number of adoptively transferred cells found in a LN 2 h after transfer and the LN size. (B) Comparison of the sizes of peripheral (brachial and inguinal) and mesenteric LNs. (C) Comparison of egress rates between peripheral and mesenteric LNs. A direct comparison between size and egress rate (like in (A) for size and entry rate) is not possible because each egress rate has to be estimated from several independent experiments whereas each entry rate is estimated from a single experiment. Data in (A) and (C) are normalized to the average entry and egress rates of CD4 and CD8 T cells, respectively, to account for the intrinsic differences between these phenotypes [35].

A quantitatively reasonable parametrization of this model is necessary to make reliable predictions. Adopting data from a previous meta-analysis of several migration experiments [17], we first set the total entry rates from blood to spleen to 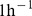, and from blood to all LNs combined to 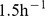. To determine the entry rates into and egress rates from the individual LNs, we analyzed raw data from a recent study [35] where adoptively transferred cells were counted in LNs at various time points after injection and LN entry blockade to estimate entry and egress rates. We found a strong correlation between LN entry rate and LN size (Figure 5A). However, egress from peripheral LNs was not significantly faster than egress from the substantially larger mesenteric LNs (Figure 5B,C), thus there is no evidence for a relationship between LN size and egress rate. This observation is consistent with the fact that large LNs are often composed of several individual lobes or compartments that each have their own entry and egress structures.

We incorporated these findings by using a single sphere to represent small LNs, and multiple spheres to represent larger LNs. These multiple spheres could for example be viewed as different lobes of an inguinal LN, or as the individual LNs that form the mesenteric LN. In our model we use 30 LNs, similar to mice [36]. We represent large peripheral LNs, i.e., brachial, axillary, and inguinal LNs, by 2 spheres each, and the mesenteric LN by 4 spheres. All other LNs (e.g., the popliteal LN) are represented by 1 sphere. We thus have 39 spheres in total. By distributing the T cells leaving the blood evenly across the spheres, and using the same sphere diameter for all LNs, we achieve that entry rate is correlated to LN size but egress rate is not. The transit time through the spheres is determined by the random walk motility coefficient and the sphere radius. We set the motility coefficient to 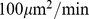, an estimate that we previously obtained from two-photon data [13], and set the sphere radius to a value that yields a physiologic average transit time of 13.5 h [17], [35]. Similarly, we set the geometric parameters of the spleen cylinder (Figure 4D) to values that lead to an average transit time of 6 h [37]. With these parameters (Table 1), the model accurately predicts a blood residence time of 25 min and a realistic distribution of T cells across SLOs (about 74% in LNs, 23% in spleen) and blood (3%; Table 2).

**Table 1:** Parameters of our two-scale migration model.

Parameter	Symbol	Value	References
LN T cell zone radius	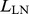	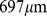	
Splenic T cell zone radius	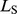	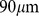	
Splenic T cell zone aperture angle	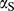	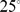	
T cell motility coefficient	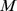	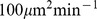	[13]
Number of LNs (*)	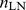	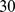	[36]
Entry rate into the 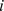 th LN in the absence of Ag	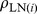	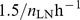	[17]
Entry rate into each dLN at day 4.5	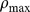	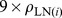	[50]
Entry rate into spleen	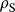	1.0 h 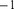	[17]

References that support the parameters are given where available. The entry rates into LNs and spleen are not experimental estimates but are adopted from earlier modeling work [17]. However, these values accurately predict the T cell distribution over the organs at steady state, the blood residence time and the blood-to-lymph transit time (Table 2). The geometric parameters (T cell zone radius in LN and spleen, aperture radius in spleen) were set to values that are both anatomically reasonable and give realistic mean dwell times in LNs and spleen. The parameters shown in this table were used for all simulation results reported in this paper unless otherwise indicated. (*) The axillary, brachial and inguinal LNs are represented by 2 spheres each, while the mesenteric LN is represented by 4 spheres. Thus, in total, we have 39 spheres.

**Table 2:** Predictions of our two-scale migration model.

Prediction	Value	References
Mean dwell time in LN	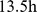	[35], [37]
Mean dwell time in spleen	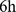	[37], [65], [66], [69], [70]
Blood residence time	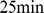	[62], [63]
T cell ratio blood:spleen:LNs	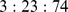	[37], [71]–[74]
Blood-to-lymph transit time	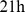	[6], [62]

Despite the simple structure of our model, its quantitative predictions (Table 2) suggest that our simulations provide a reasonable reproduction of the kinetics of T cell migration. We emphasize that this is largely achieved by setting the parameters to values reported in previous studies or derived from our own data, rather than by parameter fitting; sphere diameter and cylinder aperture angles were set to obtain realistic transit times, but the values used are anatomically reasonable. We therefore proceed to use this model to study the interplay between within-LN priming kinetics and between-LN migration kinetics.

### A trade-off between rapid arrival and robust Ag detection governs LN dwell times

Previous models [20], [21] suggested that T cell trafficking strategies have evolved subject to a trade-off: Frequently recirculating cells arrive more rapidly at relevant SLOs upon infection, but reliable Ag detection may require long dwell times within SLOs. It may seem that this conflict could be solved by letting T cells transit rapidly through non-infected LNs and keeping them longer in infected LNs. However, classic data shows that after a brief (<1d) initial “shutdown” period [38], [39], T cell egress from infected LNs is fully restored [40]–[42], perhaps to avoid that infected LNs clog up with irrelevant T cells. Hence, the baseline LN dwell time of T cells has to be long enough to ensure reliable retention of Ag specific cells, and short enough to ensure rapid arrival at infected LNs.

**Figure 6:**
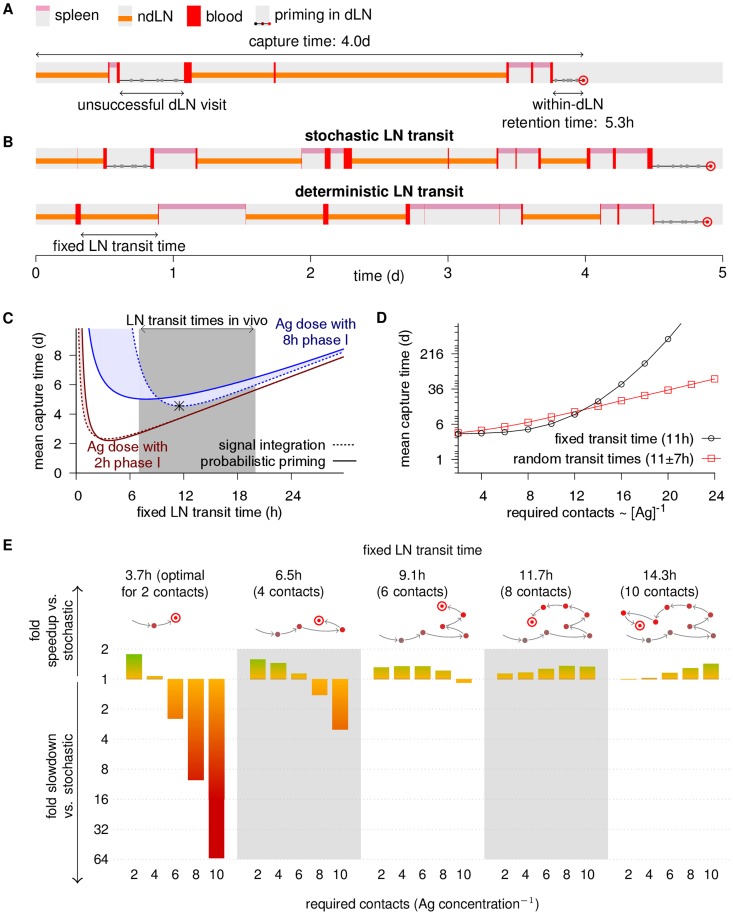
Implications of signal integration for T cell trafficking. Throughout, a hypothetical infection affecting 25% of the LNs is simulated, and the cognate DC encounter rate in LNs is 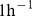. **(A)** Trajectory of a combined simulation of circulation between organs (Figure 4D) and probabilistic priming within draining LNs (dLNs) (Figure 1C). Because both migration and priming are stochastic, Ag-specific cells can egress unprimed from dLNs. **(B)** Illustration of the difference between the default stochastic LN transit (Figure 4) and simulations of deterministic LN transit, where the LN transit time is fixed. **(C)** Capture times of *in silico* cells with deterministic LN transit and signal integration or probabilistic priming. The asterisk shows the “optimal” transit time for signal integration at an Ag dose with 8 required contacts, which falls in the *in vivo* range (gray area). In contrast, the optimal transit time for a 2 h phase I is shorter than typical *in vivo* estimates (Table 1). **(D)** Capture times for stochastic LN transit with an average transit time of 11 h and standard deviation of ∼7 h compared to a fixed transit time of ∼11 h at various Ag concentrations ([Ag]). Stochastic transit is less sensitive to Ag dose variations, and has a much faster capture time for the lowest Ag concentration at 24 contacts. **(E)** Predicted benefits and risks of LN transit optimization. For various Ag doses (required contact numbers below each column), capture times of simulations with deterministic transit that would be optimal for a certain Ag dose (transit times and required contact numbers shown above column groups) were compared to the capture times of stochastic transit. Colored bars show fold differences. For example, deterministic LN transit in ∼3.7 h detects an Ag dose with 2 required contacts, for which it is optimal, almost twice as fast as stochastic transit. However, it also detects an Ag dose with 10 required contacts ∼64-fold slower.

Aiming to quantify this trade-off in our model, we combined our simulations of migration between SLOs and priming within LNs. For simplicity, we used a hypothetical infection where Ag dose and quality, as well as DC encounter rates were kept constant over time, similar to earlier models [21]. Each *in silico* cell was followed until successful retention in an Ag-bearing LN (Figure 6A). To quantify the efficiency of specific LN transit times, we also performed simulations where we let *in silico* cells spend fixed times in each LN instead of searching an exit via random walk (deterministic LN transit; Figure 6B). In each scenario, efficiency was assessed by determining the average time taken from Ag appearance until T cell retention in a LN. In the following, we refer to this time period as the *capture time* to emphasize the difference to the within-LN retention time studied above. For instance, in simulations of an infection where the Ag is present in 25% of the LN spheres, combined with signal integration priming at an 8 h phase I (like in Figure 1B), a realistic LN transit time of 12 h balances well between rapid arrival and robust retention, and leads to an expected capture time of ∼4d. In contrast, we obtain capture times of ∼6d for a transit time of 24 h and ∼9d for a transit time of 6 h (Figure 6C). Even though this quantitative prediction is based on our simplified hypothetical infection, it is intriguing to observe that the most efficient range of transit times predicted by our model is similar to physiological transit times [35].

To generalize these results, we analytically determined how the capture time depends on the migration and priming parameters (Methods), and derived equations to calculate the “optimal” LN transit time that would lead to the fastest detection of a given Ag. The results show that the disadvantage of overly long transit times is hardly affected by signal integration (Figure 6C, 24 h and beyond). However, very short transit times can be extremely detrimental with signal integration at low Ag dose (Figure 6C, 6 h and below), as most cells then exit before retention starts. With probabilistic priming, this effect is less severe as at least some cells are still retained early on.

In summary, signal integration has different implications for T cell trafficking between organs than probabilistic priming. Rapid LN transit in particular appears much less favorable when taking signal integration into account. Together with the need for T cells to receive “survival signals” by self-pMHC [21], this may explain why LN transit times are not shorter *in vivo*.

### Stochastic LN transit benefits robust Ag detection

At first sight, the prediction that LN transit time is important for rapid Ag detection appears inconsistent with the fact that T cell dwell times in LNs are widely distributed [35], [37]. If rapid capture depends on proper LN transit timing, then should evolution not have settled for a more tight control of the LN transit? For instance, it has been hypothesized that T cells migrate from the deep paracortex towards egress sites in a directed fashion [12], [43], [44]. Such a mechanism could facilitate a more precise timing of the LN transit. Indeed, if we make all cells transit every LN in the “optimal” 11.7 h instead of transiting randomly in our previous simulation (infection in 25% of the LN spheres, 8 h phase I, signal integration), the capture time slightly decreases from ∼5.5d to ∼4d – in other words, precisely timed LN transit can lead to 1.4-fold faster Ag detection in this setting. However, adjusting LN transit to optimally detect a given Ag dose comes at a price with respect to detection at other Ag doses: when performing simulations where the LN transit time was kept constant but the Ag parameters were varied, we found that T cells with deterministic LN transit were not well equipped to deal with low Ag doses (Figure 6D).

To systematically assess the potential benefits and risks of LN transit optimization, we considered Ag doses with 2 to 10 required contacts (leading to a 2–10 h phase I). For each of these settings, we computed the optimal LN transit time based on our analytical solution of the model (Methods). As expected, *in silico* cells that stay in each visited LN for exactly this optimal time detect the Ag faster than cells that transit LNs stochastically (e.g., for 8 contacts, 1.38-fold faster). However, testing how fast Ags at other doses would be detected by deterministic LN transit (e.g., we exposed cells that transit deterministically in 11.7 h, which is optimal for 8 contacts, to Ag doses requiring 2, 4, 6 and 10 contacts) showed that the risk of LN transit optimization, in terms of slower detection of “unexpected” Ag doses, can be orders of magnitude larger than the best possible gain (Figure 6E).

In reality, Ag abundance, dose, and quality will vary considerably across infections and over time. Therefore, whereas letting T cells roam freely through LNs provides robust protection against different infection scenarios, the potential speedup gained for some pathogens by tightly controlling LN transit seems to be dwarfed by the large potential risk. These findings offer an explanation for the broad distribution of *in vivo* LN residence times [35].

### Stochastic migration between organs is an effective surveillance strategy

For many infections, the immune system is able to recruit almost all Ag-specific T cells into the immune response within a few days [1], [2], which is similar to the capture times predicted by our simulations. However, these simulations were based on a hypothetical infection where Ag was instantly and constantly available, and the draining area was kept constant. Aiming for a more realistic infection simulation, we integrated data on spatial and temporal Ag availability for real infections and tested whether our model predictions are consistent with the efficient recruitment observed *in vivo*.

**Figure 7:**
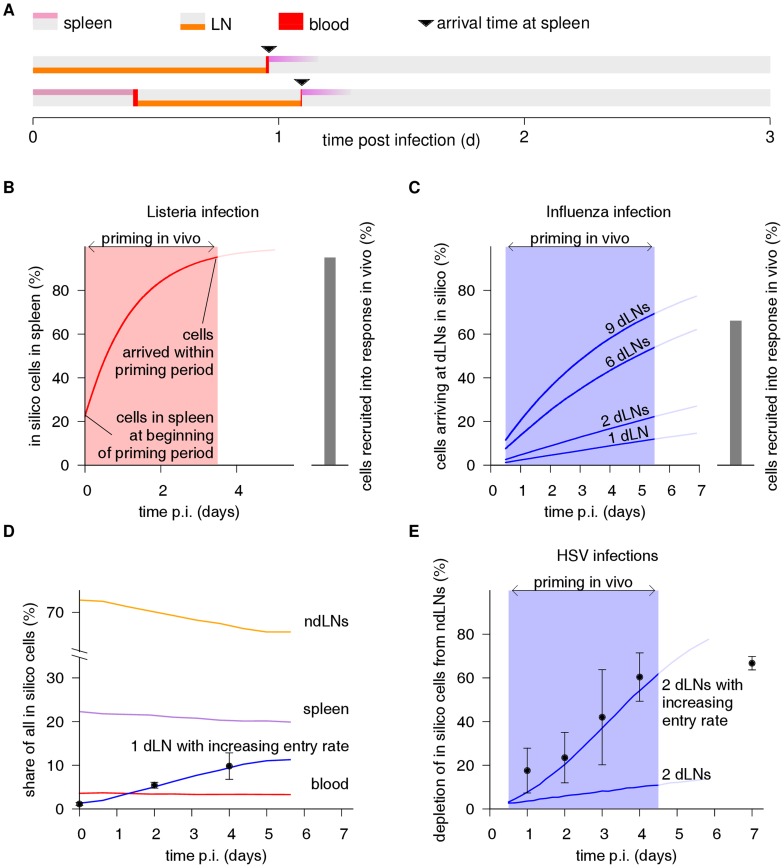
Randomly migrating T cells swiftly arrive at Ag-bearing SLOs. Throughout, dLN numbers refer to LNs represented by a single sphere. **(A)** Trajectories illustrating the simulations underlying (B) – (E): randomly circulating cells are followed until reaching an organ of interest (here, the spleen). **(B,C)** Arrival of *in silico* cells (solid lines) at (B) the spleen or (C) different numbers of dLNs during the *in vivo* priming periods (shaded areas) following (B) blood-borne *Listeria* infection [1], [46] or (C) local influenza infection [48]. The fraction of *in silico* cells that arrive during the priming period is compared to *in vivo* recruitment levels (gray bars) determined at the peak of the response [1]. **(D)** Redistribution of *in silico* cells from ndLNs, spleen and blood to 1 dLN (blue line) whose entry rate increases 9-fold during the first 4.5d p.i. compared to *in vivo* T cell numbers per dLN (circles and error bars) in vaginal HSV-2 infection [50]. T cell numbers are converted to percentages assuming that a mouse harbors 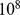 T cells [3]. **(E)** *In vivo* depletion of Ag-specific T cells from ndLNs following HSV-1 infection with 2 draining popliteal LNs (circles and error bars; ref. [2]) compared to *in silico* depletion for 2 dLNs either with (upper line) or without (lower line) increasing entry rates like in (D).

Because computation of the capture time would require detailed kinetic information on Ag dose and density as well as DC frequency and quality within LNs, we focused on the time required to *arrive at* a relevant SLO (Figure 7A). Unprimed Ag-specific cells do not egress from Ag-bearing SLOs in relevant numbers [40]–[42]. The capture time should therefore be just a few hours above the arrival time. First, we simulated arrival of Ag-specific T cells at the spleen, and found that ∼95% of all cells arrive within the first 3d (Figure 7B, solid line). We compared this prediction to data for blood-borne *Listeria monocytogenes* infections. *Listeria* enters the spleen almost instantly [45], and T cell priming then occurs mainly during the first 3–4d [46] (Figure 7B, colored rectangle). The percentage of naive T cells that are recruited into immune responses to various different infections has been estimated using cellular barcoding [1], [47]. For intravenously administered *Listeria* [1], about 95% of all Ag-specific T cells took part in the immune response (Figure 7B, gray bar). Given the arrival speed predicted by our model, a priming time window of 3.5d would suffice to support recruitment of >90% of all Ag-specific T cells even if the immune response were only formed in the spleen. Therefore, due to the large throughput of the spleen, random circulation appears sufficient for a swift response initiation against blood-borne pathogens such as *Listeria*.

Searching for Ag is more difficult for local infections, when Ag is only available in the draining LNs (dLNs) near the site of infection. Still, when applying the barcoding approach to an influenza infection in the lung, ∼2/3 of all precursors were found to be recruited into the immune response [1]. We simulated T cell arrival for different numbers of dLNs, considering T cell homing from the blood to LNs to be uniformly distributed among 30 equally sized LNs. In this setting (Figure 7C), we found that 6–9 dLNs were required to ensure arrival of 2/3 of all T cells within a typical 5d priming period of influenza [48]. This prediction is consistent with the number of LNs that drain the lung in mice – 2–8 mediastinal, 1 bronchial, and 2–4 deep cervical LNs [36]. Hence, also for local infections, randomly circulating cells can arrive at appropriate SLOs fast enough to support recruiting a majority of all Ag-specific T cells within a few days, as long as the T cell entry rate into dLNs is at least ∼20% of the T cell entry rate into all LNs.

Strikingly, it has been shown that even for an HSV-1 infection in the footpads with only 2 draining popliteal LNs, ∼2/3 of the Ag-specific T cells disappear from the circulation within 4d [2]. In contrast to the influenza infection, this result can no longer be explained by stochastic circulation alone – in that case, not even ∼20% of *in silico* cells arrive at 2 dLNs within 4d (Figure 7C). However, it is known that inflammation-driven vascular growth can rapidly and massively increase cell flux into dLNs [49], [50]. For instance, following HSV-2 infection in mice, influx increases ∼9-fold within 4d [50]. When we increase the dLN entry rate in our model accordingly (Figure 7D), a single dLN accumulates almost half as many T cells as the spleen within 3–4d, and the predicted cell disappearance from non-dLNs (Figure 7E) closely matches experimental estimates for the HSV-1 footpad injection [2].

In summary, even though our model lacks many features of T cell migration that might potentially further accelerate Ag detection and T cell removal, mere inclusion of stochastic recirculation (ensuring rapid arrival) and signal integration (ensuring reliable retention) on their own were already sufficient to explain the efficient T cell recruitment of the listeriosis and influenza case study. Only for the highly localized HSV-1 infection it was necessary to take an additional migration feature into account, namely the increased entry rate into inflamed LNs.

## Discussion

It might appear implausible that a vital function like detection of foreign Ag would depend on aimlessly wandering cells [51]. Yet, our two-scale modeling of T cell migration showed that the combination of random walk *within* tissue with stochastic migration *between* tissues is overall a very efficient and robust strategy to bring Ag-specific T cells to the correct location. Alteration of this simple migration pattern only seems necessary for local infections with very few dLNs, in which case a local increase of the dLN entry rate suffices. In fact, it turned out that stochastic migration can be superior to tightly controlled migration: Optimization of the T cell transit through LNs for most rapid detection of a particular pathogen with specific replication and Ag presentation kinetics would leave the immune system vulnerable to other pathogens, whereas stochastic transit provides far more robust protection at only slightly slower Ag detection. These results align well with the general observation that random search strategies can be very effective [52].

We aimed to base the organ representations in our model (Figure 4) as much as possible on available information on the anatomical structure of lymphoid tissue. This is in contrast to other models of T cell migration [20], [21] which instead use an exponential distribution to model egress. The main difference is that our models lead to an initial “lag period” during which no cells exit from the organ, because they need a minimum time to reach egress structures. Such a lag period might be beneficial because it prevents premature egress. However, the difference to exponential egress is not very big (Figure 4E), so our results would remain similar if we had we used a rate equation instead of explicit organ representations. Similarly, the precise shape of the compartments does not play a very big role, e.g. almost identical results are obtained when one uses a sphere to model the spleen instead of a cylinder (not shown). However, we found it reassuring to observe that realistic anatomical structures combined with realistic T cell motility lead to realistic transit times.

Our migration model does of course not capture the full complexity of T cell migration in our immune system. For this reason, we started our validation using data obtained in a carefully controlled experiment, where many of these complexities are absent. In real infections, the kinetic signature of signal integration that we aimed to detect would likely be obscured by other factors. For instance, even though statistical analysis of T cell migration in the absence of Ag does not reveal any directed migration, there could still be some directional migration hidden in the data [13], . In the presence of Ag, a weak directional bias has indeed been observed in LNs where productive interactions between CD4 T cells and DCs have already been established [55]. Such biased migration may act in conjunction with signal integration to achieve T cell retention even faster [56]. Moreover, during real infections, T cells arriving early or late at the same LN may encounter very different priming parameters. Given these complexities, we focused on the arrival kinetics when we compared our simulations to priming data for real infections (Figure 7), and stopped these simulations after arrival. Therefore, we expect the benefit of stochastic migration for robust Ag detection to be even larger in reality than our model predicts (Figure 6), given that T cells will encounter greater varieties of Ag quantity and quality *in vivo* than in our simulations.

T cell retention in LNs is thought to be mediated by upregulation of CD69, which blocks S1P-driven egress from LNs [57]. In other words, by upregulating CD69, a T cell “commits” to staying in the current LN rather than egressing and searching for Ag elsewhere. In our simulations (Figures 1 and 6), we used the onset of long-lasting stable contacts in phase II as an indicator of T cell retention. However, for low Ag dose, CD69 induction can occur already in phase I [22]. As a consequence, the capture time for e.g. an Ag with an 8 h phase I might in fact be shorter than predicted by our model (Figure 6C). Nevertheless, because we have shown that the implications of our simulations hold within a large range of within-dLN retention times, this possibility does not affect our qualitative conclusions. Moreover, it has recently been shown that in some circumstances, effector responses develop without phase II [58]. Importantly, this finding does not affect our conclusion that the onset of phase II in the data we analyzed was determined by signal integration during phase I.

Our study focused on 2 theories that explain the occurrence of short contacts at low Ag dose at the T cell level, i.e., signal integration and probabilistic priming. We found the purely probabilistic retention model, where cells do not accumulate signals from multiple interactions [14], [26], difficult to reconcile with our data. However, a further possible explanation for the transition from phase I to phase II [22], [25] could be that this is dictated by the DCs instead, e.g., as a result of ongoing DC maturation [59], [60]. Detailed information on the progress of these proposed changes at the DC level over time would be necessary to allow us to test this third hypothesis. Because such information is currently lacking, it is not possible to distinguish the DC-driven retention model from signal integration or probabilistic priming models. For the data analyzed here, however, it is hard to argue that differences in DC maturation account for the different retention kinetics, because the only change between experiments was the peptide dose, which is not known to affect DC maturation.

Our modeling results for local infections with few dLNs suggest that increased blood flow to the dLNs might be indispensable to combat such infections. This increased blood flow, and the resulting dramatic dLN enlargement, are achieved by remodeling of the central LN feeding arteriole [50], [61]. Still, even the blood flow through the enlarged arteriole amounts to only a small percentage of the cardiac blood output, and therefore it may still seem baffling how such large fractions of all T cells can arrive at the dLNs so quickly (Figure 7D,E). This finding is more easily understood when the relation between the speed of blood flow and the blood residence time is taken into account. In rodents, T cells remain in the blood for about half an hour [62], [63]. Because the cardiac output of a rodent sums up to the total blood volume within just a few seconds [64], a T cell in the blood can circulate many times through the whole body before entering an organ. Therefore, many T cells that come in close proximity of a given LN still end up homing elsewhere. Increased blood flow through the central feeding arteriole thus simply recruits a larger fraction of those lymphocytes that are passing by anyway, and a major global redistribution of the cardiac output is not required to achieve an increased entry rate into dLNs.

Although we focused on mouse data, the basic principles of our model are applicable to other species as well, including humans: The basic routes of lymphocyte recirculation described in rodents are similar to those in many vertebrates. Therefore, our qualitative conclusions likely generalize to other species. For example, also in humans with about 550 LNs we expect increased blood flow to dLNs to be extremely important for localized infections with few dLNs, and our finding that near-complete T cell retention is achievable more quickly by signal integration than by probabilistic priming is independent of the migration between SLOs. However, in species other than rodents there is still too little data on T cell migration on both scales to allow for a comprehensive quantitative analysis as we performed in this paper. In a similar vein, it was recently shown that LN dwell times differ considerably between CD8 and CD4 T cells [35]. Future work could address whether these differences might reflect different migration strategies given that these T cell subsets have very different tasks and are exposed to Ag in different contexts and interact with each other in a consequential manner.

In summary, we have presented a model of T cell immune surveillance as a two-scale stochastic search and compared the predictions of our model to various experimental findings. Even for local infections with very few dLNs, random migration between SLOs combined with a nonspecific increase of the dLN entry rate enables rare Ag-specific T cells to arrive at dLNs within a few days. Within dLNs, highly reliable retention of randomly migrating T cells can be achieved within a few hours even at low Ag densities owing to the integration of information from multiple cognate DC contacts. Overall, the two-scale stochastic migration pattern of T cells appears to be a remarkably efficient and robust solution to the needle-in-a-haystack problem of recruiting rare T cells into immune responses.

## Methods

### Two-photon data analysis

Cells were tracked using Volocity software, and statistical analysis of the cell tracks was performed using custom-written software. Tracks shorter than 2 minutes were removed from the analysis. Motility coefficients of 3D cell tracks were estimated as
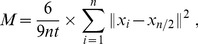
(1)


where 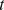 is the duration of the track, 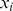 is the 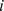
th of 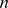 positions in the track, and 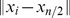 is the distance of the 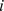
th position of the track to its middle position. For tracks of even length we use
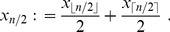
(2)


The derivation of Equation 1 is straightforward if one considers the T-cell migration as a Brownian motion. Note that in this manner we probably underestimate the motility coefficients of short T-cell tracks [13]. However, for our analysis this bias is acceptable because we do not use the actual motility coefficient values nor do we directly compare motility coefficients of cell tracks of different length.

To classify cells in a given video as retained or non-retained, we first analyze the combined set of all control cell tracks from the same experiment (several videos imaged on the same day). Let 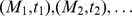 denote the motility coefficients and track durations (in video frames) of these control cells, respectively. We first compute the weighted median 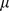 of the 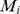, which is the median of the sequence in which each 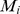 is repeated 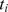 times. The threshold to define a retained cell is then set to 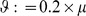. Now let 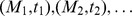 denote motility coefficients and track durations of a set of Ag-specific cells. We estimate the fraction 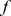 of retained cells as
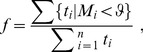
(3)


that is, the combined duration of all tracks with a motility coefficient below 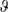 divided by the combined duration of all tracks. This way of computing 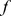 corrects for the fact that non-retained cells will have on average shorter tracks than retained cells, and therefore makes it possible to view 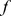 as an estimate of the fraction of retained cells simultaneously visible in the video.

### Priming models and fitting to retention data

To obtain the data for the model fitting, we consider 3 time windows of 20 min per video of 60 min length. Tracks that cross the boundary of a time window are split accordingly. Moreover, we consider the time point of each video relative to the time point of LN entry (∼1 h after injection) rather than relative to the time point of injection, because priming can only start after LN entry. From videos imaged directly after infection, we estimated that entry occurred on average 1 h after injection.

Let 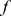 denote the fraction of retained cells in a time window estimated as described above. We correct 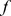 for “background noise” using the formula 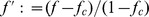 where 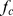 denotes the fraction of retained cells estimated by applying the above analysis to all control cells imaged in the same experiment as the given video.

In the general priming model, which combines signal integration and probabilistic priming, we consider *in silico* cells to be retained after they have established 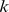 “successful” cognate encounters with 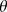 being the mean waiting time between such encounters. Because the waiting times are exponentially distributed and independent from each other, the time to retention is Gamma distributed. Hence, the function used to fit the resulting data is 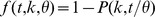, where 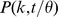 is the probability that retention 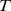 occurs before time 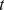, i.e. 
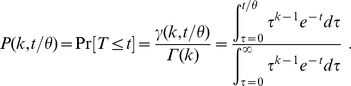
(4)


Here 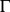 denotes the usual gamma function and 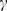 the lower incomplete gamma function. Note that our fit cannot identify the “true” DC contact rate 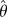 and success probability 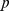, but only the rate of successful contacts 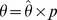: A 2 h waiting time (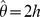) with a 50% success rate (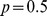) leads to 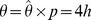 and is therefore indistinguishable from a 4 h waiting time with a 100% success rate.

When fitting the general model, we allow both 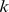 and 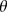 to vary between independent experiments. For instance, with 6 experiments (Figure 3), the general model has in total 12 parameters. The purely probabilistic model has 6 parameters (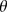 varies across experiments, 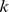 is fixed to 1) and the pure signal integration model has 7 parameters (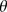 is constant across experiments, 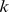 varies).

To account for heteroschedasticity (the variance of the retention data near the limits 0% and 100% is lower than near 50%), we fit the model on a logit scale. Best fits and the corresponding BIC values are computed using GNU R.

### Stochastic model of T cell circulation

Our stochastic model follows single Ag-specific T-cells. Because their frequency in the pool of all T-cells is extremely low [3], [4], Ag-specific T-cells are considered to circulate independently without influencing each others' paths. Therefore, our results are independent of the number of cells.

In the blood, *in silico* T-cells keep circulating until they encounter a random entry site into a secondary lymphoid organ (SLO). The waiting times for encountering these entry sites are exponentially distributed with rate 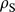 for the spleen and rate 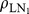 for the 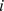
th LN sphere (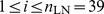; larger LNs are modelled by multiple spheres as discussed in the main text). The SLOs are modeled as three-dimensional objects. Specifically, the LN is represented as a sphere with radius 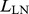. The transit of *in silico* cells through this sphere starts at the center, which represents a high endothelial venule in the LN paracortex. Cells then perform a Brownian motion with motility coefficient 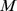 until reaching the sphere surface, which represents cortical and medullary sinusoids [12]. From the sphere surface, the cells move back into the blood. The spleen is represented as a cylinder of arbitrary length and radius 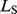. Cells enter the cylinder at a point on the left border, which represents immigration from the splenic marginal zone (Figure 1 in ref. [34]) via a marginal zone bridging channel (MZBC). They then perform a Brownian motion through the cylinder with the same motility coefficient as in LNs. However, in contrast to the LN sphere, a large part of the cylinder boundary is treated as a reflecting boundary, representing the interfaces to splenic B-cell areas that ensheathe T-cell areas. Exit is only possible through an opening on the opposite side of the entry point, which represents another MZBC and has an aperture angle of 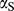. *In silico* cells reaching that opening are moved back to the blood.

The geometrical parameters of the cylinder and sphere were set to the values shown in Table 1. For the spleen, these values were set empirically based on anatomical considerations: A cylinder radius 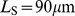 and an aperture angle 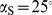 imply that a cross-section through the cylinder (Figure 4C) resembles histological PALS sections taken perpendicular to the arteriole (Figure 1 in ref. [34]). These choices lead to a mean residence time in the spleen of 6h, matching classical estimates [65], [66].

For the LN, the relationship between sphere radius, mean residence time and motility coeffcient can be analytically determined. Let 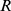 denote the residence time of an *in silico* cell with motility coefficient 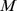 in a spherical organ of radius 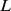. Then the expected residence time 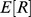 is given by 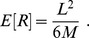



We use the estimate 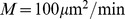, which is based on two-photon data [13], for naive T cells. Classic data indicates that in rodents, naive T cells spend on average 13.5 h in LNs [17]. Therefore, we set 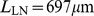. This value is anatomically reasonable for the T-cell zone of a medium-sized murine LN.

In addition to the above equation for the expected residence time, it is also possible to express the entire distribution of cell residence times in the sphere analytically [67] in terms of the infinite series 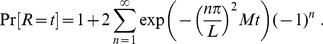
(5)


The transit time distributions for spleen and LNs in our model are shown in Figure 4E.

The model described above is easily transformed into a Monte Carlo simulation, which allows us to generate individual cell trajectories (e.g. Figure 4D) to examine the fates of simulated cells. In these simulations, cells alternate between transiting the blood and transiting an SLO. We apply the kinetic Monte Carlo method [68] to the following rate equation, which describes cell movement from the blood (B) to LNs and spleen according to the rules set out above: 
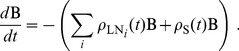
(6)


In brief, the kinetic Monte Carlo method works as follows. Let 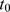 denote the time at which a cell last entered the blood, and let 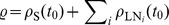 be the sum of all compartment entry rates at time 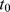. Then each organ 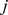 (the spleen or 1 of the LNs) is chosen as the next organ to visit with probability 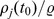. The cell is then moved to the chosen organ, and simulation time is increased by 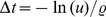, with 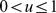 uniformly at random. The residence time of a given cell in a LN is sampled according to Equation 5, or, in some simulations (Figure 6), is set to a constant. Transit through the spleen is explicitly simulated as described above. To avoid “synchronization” between cell trajectories, Monte Carlo simulations are initialized by putting cells in the blood and letting them circulate for 1+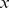 weeks, with 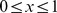 uniformly at random. After initialization, cell trajectories are recorded and the properties of interest (e.g., the arrival time at the spleen or at 1 of the dLNs) are investigated.

### Analytical solution of the capture time

The probability that a cell is retained when passing through a dLN can be expressed as 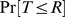, where 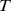 is the waiting time to retention (Equation 4) and 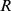 is the LN residence time (either distributed according to Equation 5 or a constant). We note that for probabilistic priming, a closed form for 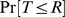 exists: Let 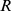 denote the residence time of an *in silico* cell with motility coefficient 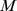 in a LN sphere with radius 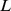. Let 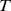 denote the retention time for probabilistic priming 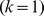 with parameter 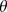. Then we have 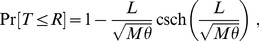
(7)


where 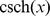 is the hyperbolic cosecant. This formula is obtained by integration.

Let us now consider the capture time 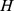 (Figure 6A). The overall efficiency of the two-scale surveillance process can be quantified by the expectation 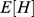 (lower 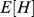 means more efficient surveillance). 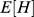 can be determined by extending our Monte Carlo simulation: When a T cell enters a dLN, the within-dLN retention time 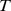 is drawn at random according to Equation 4. The T cell is considered to be retained if 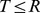. However, for simulations with constant 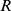 and an infection with a constant dLN entry rate and constant priming parameters 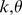 (Equation 4), 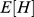 can also be determined analytically. Consider an infection starting at 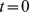, and an *in silico* T cell that is *not* in a dLN at that time. Let 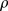 denote the fraction of dLNs among all LNs, 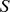 the average time spent in the blood and possibly the spleen between 2 consecutive visits of LNs, and 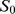 the average time at which the cell first enters a LN. Then the expected capture time is given by 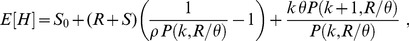
(8)


with 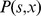 defined as in Equation 4.

Equation 8 can be obtained as follows. Let 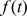 denote the probability density function of the within-dLN retention time, and let 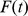 be the associated cumulative distribution function. Let the variable 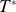 denote the time at which the cell is retained counting from the time at which it entered the *final* dLN, i.e., the dLN in which the cell eventually is retained. Let 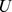 denote the number of unsuccessful visits to dLNs (that did not lead to retention) before the successful visit. Let 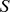 denote the time spent in blood and/or spleen between 2 consecutive LN visits, and 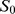 the time at which the cell first reaches a LN. Then the overall retention time 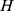 (counting from the start of the infection at 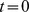) can be written as 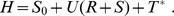



Importantly, the random variables 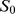, 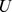, 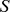 and 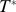 are mutually independent. For this reason, the expectation of 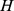 expands as follows: 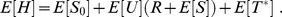



To shorten notation, we identify the variables 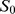 and 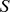 with their expectations, i.e. 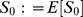 and 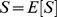. 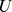 is a geometrically distributed variable with parameter 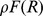. The expectation of 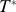 can be obtained by noting that 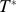 is a truncated version of the within-dLN activation time 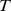, i.e., 
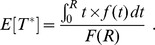



This leads to 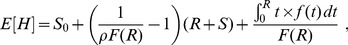
(9)


from which one obtains Equation 8 by inserting a Gamma distribution for 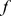 and its integral for 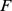.
